# Decrease in Tear Film Lipid Layer Thickness in Patients with Keratoconus

**DOI:** 10.3390/jcm11185252

**Published:** 2022-09-06

**Authors:** Wenyan Zhou, Haozhe Yu, Yun Feng

**Affiliations:** 1 Department of Ophthalmology, Peking University Third Hospital, Beijing 100191, China; 2 Institute of Medical Technology, Peking University Health Science Centre, Beijing 100191, China

**Keywords:** lipid layer thickness, keratoconus, tear film, partial blink, dry eye, meibomian glands

## Abstract

Keratoconus (KC) is a progressive corneal disorder characterized by thinning and protrusion, mostly of the inferotemporal and central corneal regions. Dysfunction of the meibomian gland, the excretions of which form the lipid layer of the tear film, has been reported to be associated with KC. Thus, this manuscript investigates the correlation among lipid layer thickness (LLT), partial blink rate (PBR), and KC of different degrees. This retrospective study included 54 patients and 24 healthy controls. The anterior corneal curvature, LLT, and PBR were taken from the unilateral eye of all 78 participants. The difference in those ocular parameters between the moderate and severe groups and the control group is significant. No significant association was found between anterior corneal curvature and LLT (r = −0.2, *p* = 0.15) across all the patients. However, there was a significant negative correlation between anterior corneal curvature and LLT in moderate (r = −0.6, *p* < 0.05) and severe (r = −0.7, *p* < 0.05) keratoconus cases. The results also show a significant negative correlation between anterior corneal curvature and PBR (r = −0.41, *p* < 0.05). Therefore, we conclude that the severity of keratoconus is associated with the thinning of LLT and the reduction of PBR. This may relate to a further epithelial abnormality with the reduced protection of tear film from the air, leading to the release of proteolytic enzymes that degrade stromal collagen and weaken the cornea.

## 1. Introduction

Keratoconus (KC) is a bilateral and asymmetrical ocular disease characterized by the thinning and ectasia of the cornea, which can lead to irregular astigmatism and significant visual impairment [1]. KC affects both genders and all ethnicities. Epidemiological studies show an incidence of 1.5 to 25 per 100,000 persons/year in the general population [2,3]. Being prevalent mainly in the second and third decades of life, the early stages of KC may go undetected and progress until the fourth decade of life. Thanks to the improvements in corneal topography and the advent of corneal tomography, corneal ectasia can be diagnosed at a much earlier stage [4]. Disease progression is accompanied by a loss of visual acuity because of the corneal curvature changes that affect light refraction [1]. Although the etiology of KC is currently poorly understood, there have been reports that multiple genes and various environmental effects are involved [3]. Besides this, oxidative stress, the downregulation of extracellular matrix proteoglycans and proteins, and decreased levels of proteinase inhibitors in the KC epithelium have also been proposed, although a molecular basis has yet to be determined [5,6]. Glasses or contact lenses are applied for vision correction in the mild and moderate stages of the disease, but surgery may be required in severe KC [7].

The tear film is an interface of about 3 μm in width between the ocular surface epithelium and the environment. Derived from the Meibomian glands, the surface lipid layer of the tear film functions as a smooth optical surface at the level of the air-aqueous interface [8]. While the thickness of the lipid layer (LLT) is only about 100 nm, it reduces surface tension and retards the evaporation of the tear film, which can reflect the function of the meibomian gland [9,10,11]. It has also been suggested that the instability of tear film has something to do with LLT and the meibomian gland [12]. Altered spreading and focal thinning of the lipid layer in Meibomian gland disease also contribute to tear instability [8]. In addition, the tear film quality is reported to result in changes in the blink rate; spontaneous blinking has complex interactions with the ocular surface [13,14].

Previous studies have reported an increased prevalence of meibomian gland dysfunction (MGD) in KC; blepharitis was found to occur more often in KC patients than in healthy individuals [15]. An association between the severity of KC and MGD has also been studied [16]. Moreover, KC patients have been reported to suffer greater tear instability [17]. Abnormal blinking could also contribute to a poor quality of vision [18]. However, the correlation between LLT, partial blink rate (PBR), and the severity of keratoconus remains unclear. Thus, this study aimed to investigate the difference between LLT and PBR between KC patients and healthy people, and the association between the severity of KC, LLT, and PBR.

## 2. Materials and Methods

### 2.1. Participants

This retrospective study was conducted at the Peking University Third Hospital in Beijing, China, from March 2018 to December 2020. The study was approved by the Institutional Ethics Committee of Peking University Third Hospital. Practices and research protocols were conducted in compliance with the tenets of the Declaration of Helsinki. Informed consent was taken from all 78 participants who were at least 18 years of age before enrollment in the study.

Exclusion criteria include a previous history of ocular surgery (i.e., glaucoma, diabetes, pterygium or contact lens wear, or cosmetics use), lid margin diseases (i.e., blepharitis, ocular allergies, irregular lid margins, ptosis, or apraxia), ocular surface diseases (i.e., dry eye disease), lacrimal apparatus diseases, conjunctivitis, keratitis, retinal pathologies, systemic diseases (i.e., systemic allergy, rheumatoid arthritis, Sjögren syndrome, or diabetes), artificial tear eye drop use, bad habits (i.e., daily screen time over 2 h, eye rubbing).

### 2.2. Clinical Classification

A total of 78 eyes from 78 patients were included in the study, including 24 healthy controls (age: 14.0 ± 8.7, male to female ratio: 1:2).

The patients were divided into three groups by the level of anterior corneal curvature according to the ABCD system of classification: the keratometry which was higher than 55.00 (including 55.00) was defined as the severe group, lower than 55.00 but higher than 48.00 (including 48.00) as the moderate group, and the left was the mild group [19].

To summarize, 54 patients were grouped into 22 patients with mild keratoconus (age: 22.3 ± 6.3, male to female ratio: 3.4), 13 patients with moderate keratoconus (age: 20.2 ± 7.2, male to female ratio: 5.5), and 19 patients with severe keratoconus (age: 21.1 ± 10.6, male to female ratio: 5.3).

### 2.3. Ocular Parameters Measurement

A single, experienced examiner measured all the average lipid layer thickness (LLT) values and partial and total blinks with the LipiView (TearScience Inc., Morrisville, NV, USA). The partial blinks rate was carefully calculated in Excel, with total blinks divided by partial blinks (Microsoft, 2018, Redmond, Washington, DC, USA).

Another expert examiner measured all the anterior corneal curvature measurements using the Pentacam (Optikgrate GmbH, Wetzlar, Germany).

Table 1 presents the numerical data of the basic characteristics of all the participants.

### 2.4. Statistical Analysis

The statistical analysis was performed using R 4.0.4 software. The results obtained and statistically analyzed were expressed with mean ± standard deviation (SD). Quantitative data were expressed using the mean ± standard deviation and compared by general linear model, while qualitative data were expressed using ratios. Spearman’s rank correlation analysis was used for exploring the association between Lipiview-related parameters with anterior corneal curvature. A *p*-value of less than 0.05 was considered to be statistically significant.

## 3. Results

### 3.1. Basic Clinical Features of Patients

**Figure 1:**
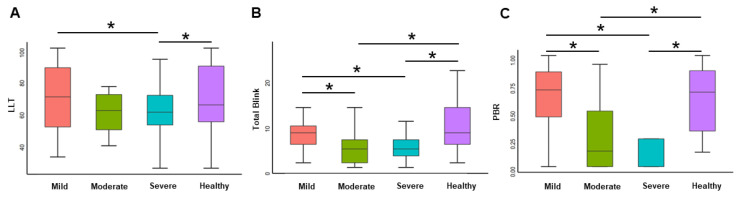
Characteristics of LLT (**A**), total blink (**B**), and partial blink rate (**C**) of subjects. (* *p* < 0.05)

Figure 1 presents the ocular surface-related parameters, measured by LipiView, of all subjects. The LLTs of healthy controls and mild keratoconus patients were similar, and both were greater than severe patients; however, the difference with moderate patients was marginally significant (*p* < 0.1) after adjusting for age and the male/female ratio. The total blink and partial blinking rates (PBR) were also similar in healthy controls and patients with mild keratoconus. Still, the rates were higher than those in patients with moderate and severe keratoconus, while the difference between the latter two was not statistically significant.

### 3.2. Association of LLT and Anterior Corneal Curvature

**Figure 2:**
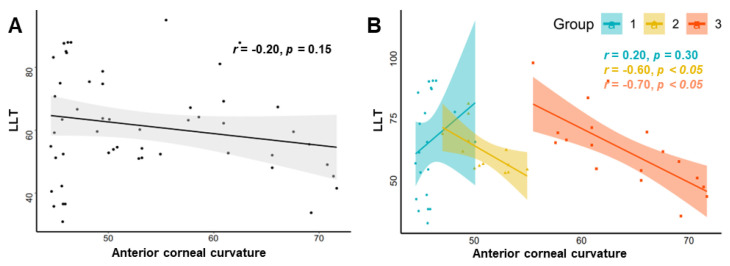
Partial correlogram of LLT and anterior corneal curvature. (**A**) Correlation of the overall KC patients. (**B**) Correlation of the KC patients with different severity.

Figure 2A shows the correlation between the anterior corneal curvature and LLT after adjusting for age and male/female ratio. However, no significant correlation was found across the entire curvature range. This correlation was further explored according to the different severity of keratoconus (Figure 2B). It can be observed that for moderate and severe keratoconus, there was a significant negative correlation between these two parameters, while no difference was found between the correlation coefficients. Notably, for the mild keratoconus group, LLT showed a positive correlation with anterior corneal curvature; however, the correlation coefficient was not statistically significant.

### 3.3. Association of PBR and Anterior Corneal Curvature

**Figure 3:**
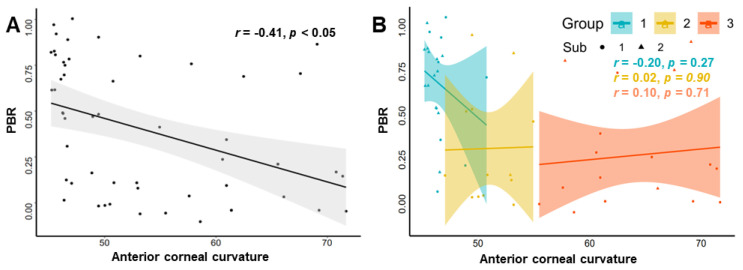
Partial correlogram of PBR and anterior corneal curvature. (**A**) Correlation of the overall KC patients. (**B**) Correlation of the KC patients with different severity. Sub 1, 2 represent total blink rates of <8 and >8, respectively.

Figure 3A demonstrates a significant negative correlation between anterior corneal curvature and PBR after adjusting for age and the male/female ratio, while the association disappeared when grouped according to the severity of keratoconus (Figure 3B). In addition, the distribution of PBR appeared to be discrete; therefore, the subgroup analysis of PBR was further performed according to the total blinks. It can be seen that the vast majority of patients with lower total blinks (<8) generally demonstrated lower PBR, whereas patients with high total blinks generally have higher PBR. The statistical significance of such phenomena was confirmed in patients with severe keratoconus (*p* < 0.05).

## 4. Discussion

The main finding of this study was a significant negative correlation between relatively severe keratoconus and LLT, as expected, although no difference was found across the entire curvature range. The results also showed that there was a significant negative correlation between anterior corneal curvature and PBR after adjusting for age and the male/female ratio. Besides this, in patients with severe keratoconus, we found that total blinks were positively associated with partial blink rates (PBR).

According to the baseline statistics, the LLT of healthy controls and mild keratoconus patients were both more noteworthy than in severe patients, despite the fact that the former two were similar. Notably, for the mild keratoconus group, LLT showed a positive correlation with the anterior corneal curvature, although the correlation coefficient was not statistically significant. Thus, we hypothesized that a compensation function of the meibomian gland may occur in the early stage of KC to stabilize the tear film [8]. In addition, there have been reports that a gradually significant increase in corneal astigmatism was found alongside the progressive stage of the KC [20,21]. Intensified ectasia of the cornea may contribute to focal thinning of the lipid layer.

In our study, we found a negative association between KC in severe cases and LLT. Although no specific studies have yet been published on the association between KC and LLT, the correlation between KC and the meibomian gland has been reported. Meibomian gland dysfunction was found to be more prevalent in the more severe cases of KC [16]. Besides this, a significant difference in gland distortion between the different stages of KC is also reported; the meiboscore correlated well with the KC staging [22]. Assuming the fact that the meibomian gland influences LLT and the stability of tear film, our findings are in line with those studies.

Dry eye syndrome (DES) is a common chronic multifactorial condition of the ocular surface; more than 80% of patients have the hyper-evaporative type, which is related to meibomian gland dysfunction (MGD) [23]. A pilot study of the concentrations of Ap4A in human tears, which increase concomitantly with dry eye and are an objective marker for DES, showed that levels were 20 times higher in KC patients than those of healthy subjects [17]. As a result, a few investigations have proposed that keratoconus might share a similar mechanism with DES, both of which have a negative impact on the meibomian gland [24]. Besides this, DES is more common in KC patients than in healthy controls [16,17]. However, a previous study shows a high level of variability in the relationship between the DES and LLT, though a significance between the two might exist [25]. Other studies report that LLT and DES symptoms do not have a linear relationship [26]. In addition, a study of DED patients showed that increased age was positively associated with LLT [27]. The average age of our patients is between twenty and thirty, and we have adjusted for age. Therefore, our explanation is, that compared to DES, KC may depend more on a qualitative alteration of the tear film wherein the LLT becomes thinner, caused by meibomian gland dysfunction, rather than because of a quantitative defect. Thus, the qualitative alteration of the lipid layer may be a dangerous factor to increase the levels of proinflammatory chemokines, as it has been said that an imbalance between the cytokines (pro-inflammatory and anti-inflammatory) may lead to altered corneal structure and function [28].

Blinking can retain the dynamic balance of ocular surface tear capacity and is essential for the development and distribution of the lipid layer [29], while partial blinks were defined as blinks without touching the upper and lower eyelids [30]. Previous studies showed that partial blinks contributed to the change in tear film stability. Owing to the increase in partial blinking, the inferior ocular surface will suffer inadequate lipid distribution, which may increase evaporation [29,31]. Recently, it has been reported that partial blinking rate (PBR) also significantly correlates with meibomian gland dropout. In our study, the PBR of the healthy control and mild symptoms groups is much greater than the two relatively severe KC groups. We also found a significant negative correlation between anterior corneal curvature and PBR, while the association disappeared when grouped into the severity of keratoconus. It indicated that partial blinks remained unchanged, along with the development of keratoconus. A contralateral eye study reported that blinking is an action associated with consciousness and that this behavior is difficult to alter within a short time [32]. This may help to explain the phenomenon. In addition, we found that the total blink rate was greater in healthy controls than in severe groups, which means that the decrease in PBR with development in KC indicates a decrease in partial blinks. The diminished amount of collagen, as well as a reduction in the interfibrillar distance of collagen of the cornea, has been reported in KC [33]. We speculate that the thinning of the cornea makes it easier to blink completely each time, although the intervals between blinks may be prolonged.

However, until now, there has been no report of the correlation between PBR and keratoconus. Studies were focused on the association between LLT and PBR and most did not find a correlation between the two. In a study of nasolacrimal duct obstruction patients, the blinking dynamics produce a difference in LLT after silicone tube intubation, although the ratio of partial and total blinking may not affect the LLT [34]. Similarly, no statistically significant difference was observed between LLT and the total and partial blink rates over a set time period in both groups of DES patients after surgery [35]. One explanation of our results is that during the examination of the partial blink using Lipiview, there is a strong flicker that could disturb the patient’s natural blink rate [32]. Another is that these patients may blink less with long-term glasses-wearing and computer use [36,37].

This study is burdened by several limitations. First, this is a retrospective cross-sectional study. Our sample size is relatively small; thus, we are not able to draw causal conclusions from the results. In addition, we did not collect the parameters of the meibomian glands of our participants. However, we focused mainly on the correlation between LLT and KC; the meibomian gland is only a potential influencing factor of LLT and we will further explore the findings in the future.

In conclusion, we found that LLT, total blink rate, and PBR were much greater in the control group than in severe KC patients, but there is no difference between healthy controls and mild KC patients among those three parameters. Besides this, a negative association between keratoconus and LLT was found, especially in severe cases, and a lower partial blink rate was found to relate to more severe keratoconus. We think that the detection of LLT can be used as an aid for clinicians to diagnose KC.

## Figures and Tables

**Table 1:** Baseline characteristics of the enrolled subjects.

Group	Age	Male/ Female	Front K	LLT	Total Blink	PBR
Healthy controls	14.0 ± 8.7	1.2	-	68.1 ± 4.6	10.1 ± 1.3	0.60 ± 0.06
Mild keratoconus	22.3 ± 6.3	3.4	46.5 ± 0.3	67.5 ± 4.6	9.0 ± 1.1	0.63 ± 0.06
Moderate keratoconus	20.2 ± 7.2	5.5	51.0 ± 0.7	59.5 ± 3.3	5.6 ± 1.1	0.26 ± 0.09
Severe keratoconus	21.1 ± 10.6	5.3	63.9 ± 1.3	60.4 ± 4.3	5.5 ± 0.7	0.22 ± 0.07

## Data Availability

The data presented in this study are available on request from the corresponding author.
